# Arsenic Exposure and Outcomes of Antimonial Treatment in Visceral Leishmaniasis Patients in Bihar, India: A Retrospective Cohort Study

**DOI:** 10.1371/journal.pntd.0003518

**Published:** 2015-03-02

**Authors:** Meghan R. Perry, Vijay K. Prajapati, Joris Menten, Andrea Raab, Joerg Feldmann, Dipankar Chakraborti, Shyam Sundar, Alan H. Fairlamb, Marleen Boelaert, Albert Picado

**Affiliations:** 1 Division of Biological Chemistry and Drug Discovery, College of Life Sciences, University of Dundee, Dundee, Scotland, United Kingdom; 2 Department of Biochemistry, Central University of Rajasthan, Bandarsindri, Kishangrah, Ajmer, Rajasthan, India; 3 Infectious Disease Research Laboratory, Department of Medicine, Institute of Medical Sciences, Banaras Hindu University, Varanasi, India; 4 Department of Public Health, Institute of Tropical Medicine, Antwerp, Belgium; 5 College of Physical Sciences—Chemistry, Trace Element Speciation Laboratory, University of Aberdeen, Aberdeen, Scotland, United Kingdom; 6 School of Environmental Studies, Jadavpur University, Kolkata, India; 7 ISGlobal, Barcelona Centre for International Health Research (CRESIB), Hospital Clínic—Universitat de Barcelona, Barcelona, Spain; Institute of Tropical Medicine, BELGIUM

## Abstract

**Background:**

In the late twentieth century, emergence of high rates of treatment failure with antimonial compounds (SSG) for visceral leishmaniasis (VL) caused a public health crisis in Bihar, India. We hypothesize that exposure to arsenic through drinking contaminated groundwater may be associated with SSG treatment failure due to the development of antimony-resistant parasites.

**Methods:**

A retrospective cohort design was employed, as antimony treatment is no longer in routine use. The study was performed on patients treated with SSG between 2006 and 2010. Outcomes of treatment were assessed through a field questionnaire and treatment failure used as a proxy for parasite resistance. Arsenic exposure was quantified through analysis of 5 water samples from within and surrounding the patient’s home. A logistic regression model was used to evaluate the association between arsenic exposure and treatment failure. In a secondary analysis survival curves and Cox regression models were applied to assess the risk of mortality in VL patients exposed to arsenic.

**Results:**

One hundred and ten VL patients treated with SSG were analysed. The failure rate with SSG was 59%. Patients with high mean local arsenic level had a non-statistically significant higher risk of treatment failure (OR = 1.78, 95% CI: 0.7–4.6, p = 0.23) than patients using wells with arsenic concentration <10 μg/L. Twenty one patients died in our cohort, 16 directly as a result of VL. Arsenic levels ≥ 10 μg/L increased the risk of all-cause (HR 3.27; 95% CI: 1.4–8.1) and VL related (HR 2.65; 95% CI: 0.96–7.65) deaths. This was time dependent: 3 months post VL symptom development, elevated risks of all-cause mortality (HR 8.56; 95% CI: 2.5–29.1) and of VL related mortality (HR 9.27; 95% CI: 1.8–49.0) were detected.

**Discussion/Conclusion:**

This study indicates a trend towards increased treatment failure in arsenic exposed patients. The limitations of the retrospective study design may have masked a strong association between arsenic exposure and selection for antimonial resistance in the field. The unanticipated strong correlation between arsenic exposure and VL mortality warrants further investigation.

## Author Summary

The parasitic disease visceral leishmaniasis (VL) causes a significant burden of illness and death in India. The main drug used to treat VL, which is based on the chemical element antimony, stopped working well in about half of all patients in the late twentieth century. We hypothesised that arsenic exposure of the Indian population, through contaminated groundwater, was contributing to treatment failure with antimony based drugs. Arsenic and antimony are similar chemical elements and exposure of the parasite to arsenic within the liver of arsenic-exposed patients could allow the parasite to become resistant to treatment with antimony. Using a field-based questionnaire study we retrospectively evaluated whether arsenic exposure was linked to antimonial treatment failure in a cohort of 110 antimonial treated patients. No significant association was found, although this may be because the number of patients in the study was low as antimony use was officially discontinued in 2005 due to high rates of treatment failure. However, arsenic exposure was found to increase risk of mortality from VL particularly if death occurred more than 3 months after the symptoms of VL developed. More research into the relationship between arsenic exposure and mortality in VL is warranted.

## Introduction

In the late twentieth century, the emergence of high rates of treatment failure with antimonial compounds for visceral leishmaniasis (VL) caused a public health crisis in Bihar state, India [1]. VL, also known as kala-azar, is a significant health problem in India causing up to 282,000 clinical cases per year [2], affecting the most vulnerable populations and leading to significant morbidity, mortality and economic loss [3]. VL is a potentially fatal disease that is caused by the obligate intracellular parasite *Leishmania donovani* transmitted by the bite of female sand flies of genus *Phlebotomus argentipes*. In India, VL is diagnosed through visualization of the parasites on splenic or bone marrow biopsy or through rapid diagnostic tests (e.g. a dipstick detecting antibodies against rK39) [4].

Pentavalent antimonial compounds such as sodium stibogluconate (SSG) had been the predominant successful treatment against VL during the twentieth century and, in endemic regions such as East Africa, they remain integral and effective in treatment regimens [5,6]. In India, since 2005, SSG use has no longer been officially recommended due to high rates of treatment failure. The VL elimination program in India has been using miltefosine and amphotericin B [7] as an alternative to SSG. However field studies have shown that, if stocks are available, SSG is still prescribed [8]. The reason for the dramatic decline in the efficacy of antimonials in India has previously been attributed to the development of drug resistance in parasites in a context of poor prescribing practices and irrational drug use in India’s under-regulated and largely private health care system [9]. An additional hypothesis has recently been put forward by our group: that background exposure of the population of Bihar through drinking arsenic-contaminated groundwater may have contributed to the development of antimony-resistant parasites [10].

Arsenic and antimony are elements that share many common chemical properties [11] including occurrence in nature in the trivalent and pentavalent states. *Leishmania* promastigotes resistant to trivalent antimony can be created in the laboratory through stepwise increasing exposure to trivalent arsenic [12]. Naturally occurring arsenic contamination is present in the groundwater accessed by tube wells in many villages of Bihar and, although this was not discovered until 2002 [13], villagers may have been drinking arsenic contaminated water from tube wells that were first sunk in the region in the late 1970s. It is thus possible that parasites were exposed to accumulated arsenic in the liver and spleen of populations drinking arsenic-contaminated water, and became cross-resistant to antimony through mechanisms developed to deal with arsenic. Proof of concept of this hypothesis has been established in our laboratory by exposing mice to environmentally relevant levels of trivalent arsenic in their drinking water and demonstrating diminished efficacy of the pentavalent antimonial compound SSG in vivo and, in an in vitro macrophage assay, that *ex vivo* amastigotes from these arsenic exposed mice were resistant to 500 μg/ml SSG [14]. The mode of action of pentavalent antimonial compounds is through activation to their toxic trivalent form resulting in disruption of the thiol metabolism of the parasite [15], mechanisms which are thought to be disabled in antimonial resistant parasites [9].

Compiling information from published articles, surveys and databases, we identified 10 out of 38 districts in Bihar where VL, arsenic contamination of the groundwater and antimonial treatment failure co-exist [10]. Unfortunately, monitoring of clinical outcomes of VL treatment in this region is sub-optimal [8] and there have been no population-based surveys done here on arsenic contamination so there may be many more districts in Bihar affected by the same conditions.

Having established proof of concept of accumulated arsenic inducing pentavalent antimony resistance in vivo in the laboratory, we sought to test the hypothesis that arsenic exposure increases the risk of VL treatment failure with antimonial compounds in the field setting. This presented a number of challenges in study design. First, the study was retrospective, since SSG is no longer widely used in Bihar. Second, it was not possible to test parasite isolates for SSG susceptibility from patients treated years previously. Consequently, treatment failure was used as a proxy for parasite resistance. Although treatment failure can be a multifactorial event, exposure to arsenic varies within the study area. Thus, the primary objective of this study was to evaluate if arsenic exposure is related to treatment failure in SSG-treated patients in Bihar, India. The secondary objective was to evaluate if arsenic exposure is related to death from VL in this antimonial treated cohort.

## Methods

### Ethics statement

Ethical clearance was obtained from Banaras Hindu University in Varanasi, the Kala Azar Medical Research Center (KAMRC) in Muzaffarpur, India and the University of Dundee, Scotland. Individual written consent was obtained from each patient (or their guardian) included in the study. Any person identified with symptoms of VL or post kala-azar dermal leishmaniasis (PKDL) during the study was referred to an appropriate health care facility. Data from the arsenic analysis of tube well samples was referred to the Public Health and Education Department, which is running the arsenic mitigation programme in Bihar.

### Study area

The study took place in Mohiuddin Nagar block in the Samastipur district of the state of Bihar, India. Bihar is the third largest state in India and has the lowest literacy rate. Mohiuddin Nagar block lies just north of the River Ganges and has a mainly rural population of 184,521 [16]. It is known to be endemic for VL; between 2006–2010, the average reported yearly incidence of VL was 7.78 per 10,000 population (Source: District Malaria Office records, Patna, Bihar) and Samastipur district, in which it lies, has been mapped as an area with ‘high’ levels of resistance to SSG [17]. A survey performed by the School of Environmental Studies, Jadavpur University in 2005 identified over 40% of the wells surveyed in this district to have arsenic levels above the World Health Organization (WHO) recommended limit of 10 μg/L [18].

### Study design and population

The fact that SSG treatment for VL is no longer recommended for routine use in Bihar [19] precluded analyzing parasites isolates from patients treated with SSG to determine in vitro resistant phenotypes. To overcome this limitation we designed a retrospective cohort study, using treatment failure as a proxy for presence of parasite resistance. We identified VL patients treated with SSG between 2006 and 2010 in the block of Mohiuddin Nagar from two sources. First, in January and February 2012, potential SSG treated subjects were identified from the VL patient register at the Primary Health Centre (PHC) in Mohiuddin Nagar and were searched for in their listed villages, at least twice, for inclusion in the study. Second, additional VL patients were identified in the visited villages. The patients were included in the study if they gave a clear history of having been treated with SSG (see below) and at least one of the SSG treatments fell inside the study recruitment period (e.g. 2006 to 2010). Patients were excluded if they did not receive SSG treatment or if their SSG treatment was terminated due to unavailability of SSG.

The study subjects were visited in their own home. A modified form of a previously validated questionnaire was used [8] to gather information on age, sex, caste, VL symptoms, health seeking behaviour, treatment(s) and response from the time of first onset of VL symptoms to the time of the field study. Additional data was collected on other illnesses, prior VL in the family and water sources used (e.g. local tube wells). The interviews were performed by experienced field workers. If the patient was a minor at the time of VL, his/her guardian was interviewed and if the patient had passed away or was unavailable due to relocation for marriage or work, then a relative was interviewed instead. The household tube well and local tube wells were geo-located by the research team using a GPS. The household tube well coordinates were used to identify patients’ location (e.g. within or outside Mohiuddin Nagar town).

Clinical records, either the patient’s own documents or those held at the PHC, as well as data gathered during the interviews were reviewed by an experienced physician to ascertain VL cases and treatment. In absence of treatment records, the information provided by patients or relatives allowed identifying the type of treatment received as the 3 main drugs for VL are given via different routes: (1) SSG is an intramuscular injection usually administered for 30 days in the lateral upper thigh or buttock area; (2) amphotericin B is given via an intravenous drip in the forearm or hand for either 5 days or 30 days dependent on formulation used and (3) miltefosine is administered for 28 days as a capsule for oral use.

Patients were classified into the following treatment outcome categories: success, no clinical improvement, relapse, death and toxicity taking 6-month cut off time from end of treatment as reference, in accordance with WHO guidance and VL clinical trial protocols [1]. ‘Treatment success’ was defined as patients who received SSG treatment and had not required another VL treatment within 6 months. ‘No clinical improvement’ were patients who experienced no change or a worsening of their original symptoms during or by the end of a full course of treatment who required a further VL treatment. This subgroup included patients who reported a treatment course of 60 days or more which is 2 times the recommended SSG treatment duration. ‘Relapse’ were patients who experienced a return of signs or symptoms of VL, for which they required further treatment, after initially having experienced a return to health following a course of treatment. If the outcome was ‘Death’, a verbal autopsy was carried out by the physician on the field team to ascertain if the subject died directly as a result of VL. ‘Toxicity’ were patients whose treatment was terminated due to intolerable side effects. The term ‘treatment failure’ in this study covers all adverse outcomes that occurred within 6 months of VL treatment: no clinical improvement, relapse, death due to VL and toxicity.

### Arsenic exposure

The average arsenic concentration of the patient’s main water source and 4 tube wells surrounding the patient’s home was taken as an indication of the level of arsenic exposure for the patient. This exposure variable was chosen instead of the arsenic concentration in the patient’s primary tube well [20] in the context of this study as many patients reported that their current primary tube well had been inserted in the years after their first SSG treatment for VL.

At the time of interview samples were collected from these 5 tube wells in a 15 ml Falcon tube after pumping off water for two minutes. Water samples were preserved with a drop of concentrated nitric acid until analysis. Flow injection hydride generation—atomic absorption spectrometry (FI-HG-AAS) was used to quantify the total arsenic content in water samples at the School of Environmental Studies (SOES), Jadvapur University, Calcutta as described previously [13]. A standard sample from the Environmental Protection Agency was used as a reference. The lower detection limit was 3 μg/L of arsenic. For quality control, 25% of the primary tube well water samples were selected to represent the low (n = 6 (<10 μg/L)), medium (n = 15 (10–50 μg/L)) and high (n = 9 (>50 μg/L)) arsenic concentrations detected. These samples were re-analyzed by Inductively Coupled Plasma Mass Spectrometry (ICP-MS) at Aberdeen University using a method described previously [21].

### Data management

The average arsenic concentration in the 5 tube wells was dichotomized using the WHO threshold for arsenic (As) in water: > = 10 μg/L. The three outcome variables are: (1) SSG treatment outcome (primary outcome) and (2) all-cause and (3) VL mortality (secondary outcomes). For the primary analysis, VL patients were classified based on their SSG treatment outcome as “treatment failure” or “treatment success” as described above. For the secondary mortality analyses, the study subjects were classified as “alive” or “dead” at the time of the field study. A sub-group identifying those that died due to VL was created based on the data from the verbal autopsies (see above).

Data from the field interviews were double entered in a Microsoft Excel database independently by 2 data entry operators. The covariates included in the analyses are shown in Table 1. Briefly, age was split into 3 categories (e.g. < 5, 6–15 and > 16 years old) and SSG treated patients were dichotomized based on their “treatment course” using the duration of 30 days recommended by WHO [5]. Patients were classified based on their location (e.g. in or outside of Mohiuddin Nagar town), the “time to treatment” (e.g. < 12 weeks and ≥ 12 weeks in accordance with previous literature [22]) and “the place of treatment” (e.g. government or private facilities). Finally, patients were classified into whether they had family members treated for VL with SSG prior to the patients’ VL episode.

**Table 1:** Bivariate logistic regression analysis of risk factors for SSG treatment failure.

Variable	Treatment failure	Treatment success	OR (95% C.I.)	p-value
	n = 62* (%)	n = 48 (%)		
**Sex**				
Female	26 (41.9)	17 (35.4)	0.76 (0.35–1.65)	0.488
Male	36 (58.1)	31 (64.6)		
**Age**				
0–5	9 (14.5)	6 (12.5)	1	
6–15	25 (40.3)	16 (33.3)	1.04 (0.31–3.49)	0.947
>15	28 (45.2)	26 (54.2)	0.72 (0.22–2.30)	0.576
**Location**				
Outside MHD town	49 (79.0)	40 (83.3)	1.32 (0.50–3.52)	0.570
MHD town	13 (21.0)	8 (16.7)		
**Caste**				
General upper class	8 (12.9)	4 (8.3)	1	
Backward class	37 (59.7)	29 (60.4)	0.64 (0.17–2.33)	0.496
Schedule lower class	17 (27.4)	15 (31.3)	0.57 (0.14–2.27)	0.422
**Previous VL treatment with SSG in family**				
No	49 (80.3)	44 (91.7)	2.70 (0.81–8.97)	0.106
Yes	12 (19.7)	3 (8.3)		
**Time to treatment, weeks**				
0–12	48 (77.4)	39 (81.3)	1.26 (0.49–3.23)	0.625
>12	14 (22.6)	9 (18.8)		
**Place of treatment**				
Private	8 (12.9)	8 (16.7)	1.35 (0.47–3.90)	0.580
Government	54 (87.1)	40 (83)		
**Full duration of treatment with SSG (30 days)**				
No	33 (53.2)	14 (29.2)	0.36 (0.16–0.80)	0.012
Yes	29 (46.8)	34 (70.8)		
**Mean local arsenic level (tube wells, n = 5)**				
<10 μg/L	45 (72.6)	39 (81.3)	1.64 (0.66–4.09)	0.291
≥10 μg/L	17 (27.4)	9 (18.7)		

Note: *This number includes 10 deaths.

### Statistical analyses

Dot plots of the log of arsenic exposure were drawn for the outcomes (1) SSG treatment outcome, (2) all-cause mortality and (3) VL mortality and the median arsenic exposures were compared using Mann Whitney U test. Receiver Operating Characteristic (ROC) curves were used to evaluate the WHO cut off for arsenic in water (10 μg/L) against the primary and secondary outcomes (e.g. SSG treatment, all-cause and VL mortality). The Kappa index was used to evaluate the agreement between the arsenic levels reported by the two laboratories (SOES and Aberdeen University) that analyzed the quality control samples.

A logistic regression model was employed to assess if arsenic contamination in the local environment increased the risk of treatment failure in SSG treated patients. First, each covariate was analyzed against the main outcome using logistic regression. Then, a multivariate logistic regression model was built, using a forwards stepwise method, with three a priori selected variables (“forced” variables: age, sex and location). Any additional variables which had a p value of <0.2 in the bivariate analyses were evaluated in the multivariate model and retained if they had a p value of < 0.05. The results were presented as odds ratios (OR) and their 95% Confidence Intervals (CI).

Survival analysis methods were used to compare survival in subjects exposed to elevated (> = 10 μg/L) versus normal arsenic water levels. The time origin for the survival analysis was the reported start date of symptoms. The date of 01/02/12, mid date of field visits, was used as the censor date. First, a Kaplan Meier (KM) survival curve and the log-rank test were used to evaluate the association between arsenic in water and all-cause mortality. Cox regression was used to estimate the risk of all-cause mortality in patients exposed to arsenic contaminated wells while controlling for possible confounding factors. The proportional hazards assumption was tested using visual methods and on the basis of scaled Schoenfield residuals [23]. If the proportional hazard assumption was invalid the effect of arsenic exposure was allowed to vary over time by fitting 2 time varying covariates in the model. The KM plots were used to determine the temporal periods in which the effect of arsenic changed. The final regression model included arsenic exposure, three a priori selected variables (“forced” variables: age, sex and location) and any variables that were associated with mortality as assessed by the log rank test (p <0.2) and remained significant (p <0.05) in the final model. Hazard ratios (HR) and their 95% CI were used to express the risk in the final model. Analogous analyses (e.g. KM and Cox regression model) were used to study the risk of VL mortality associated with arsenic exposure.

## Results

### Study population

**Fig 1:**
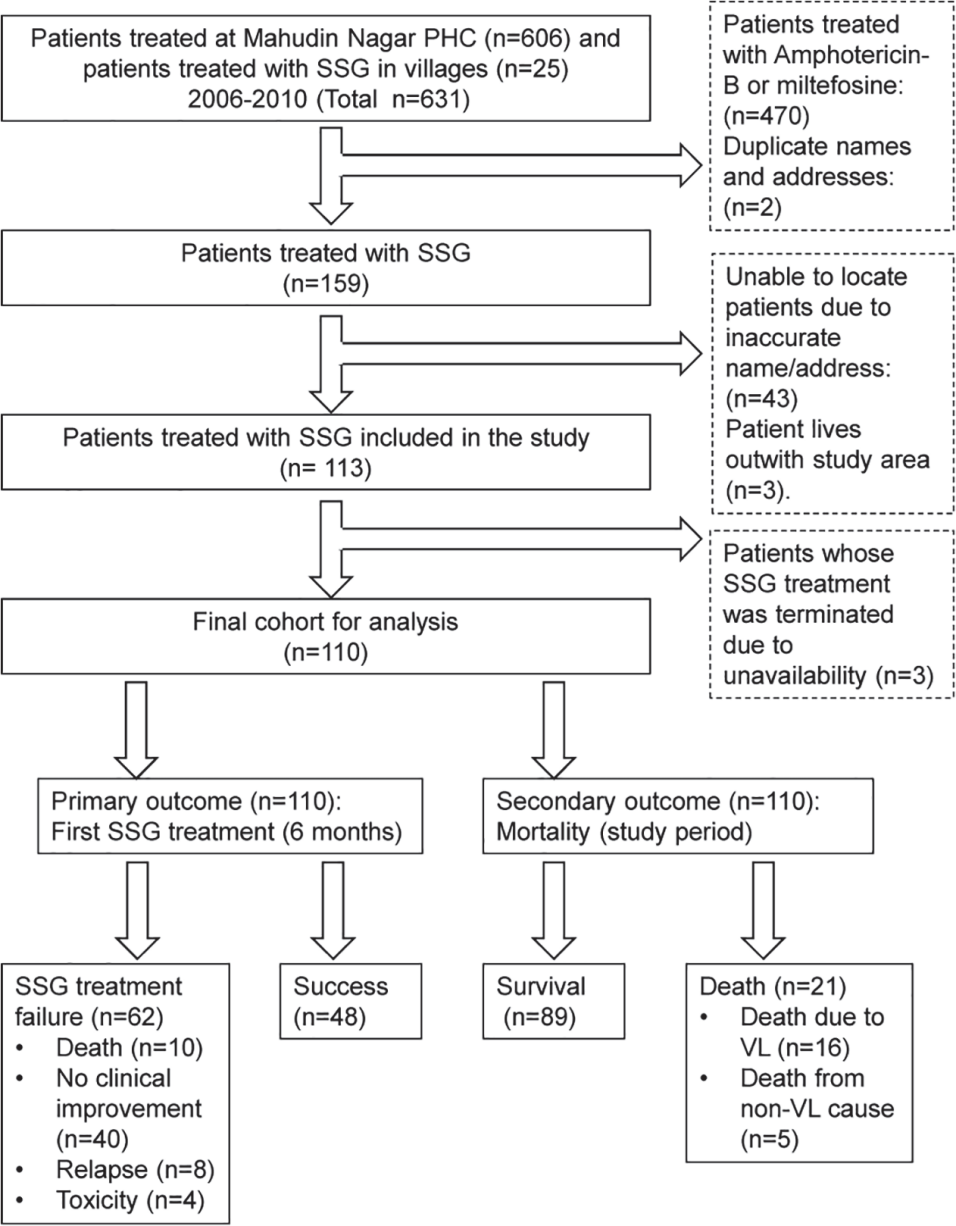
Flow chart of individuals identified as having received SSG treatment between 2006 and 2010. Additional outcomes: One patient had a still birth during antimonial treatment. One patient was identified with PKDL at the time of study.

We identified 606 patients treated for VL from January 1st 2006 to December 31st 2010 at the PHC of Mohiuddin Nagar. One hundred and thirty four (22%) of them were treated with SSG. The rest (n = 472) were excluded from the study as they received miltefosine or amphotericin B (n = 470) or were duplicate entries (n = 2). Twenty-five additional VL patients treated with SSG from 2006 to 2010 were identified at the time of the fieldwork visits. Out of the 159 SSG treated patients identified, the house of 113 (71%) of them was located. The reasons for being unable to locate patients included incomplete/inaccurate information (n = 43) and distance from Mohiuddin Nagar block (n = 3). From the 113 patients, 69 (61%) of them were present for interview. Twenty one (19%) of the subjects were dead and 23 (20%) were living outside the study area at the time of the visit. Relatives from the 44 missing subjects were interviewed. Based on the information gathered, 3 patients were excluded as they had their SSG treatment terminated due to unavailability of the drug. A cohort of 110 VL patients treated with SSG was finally included in the analyses (Fig. 1).

### VL cases and treatment

**Fig 2:**
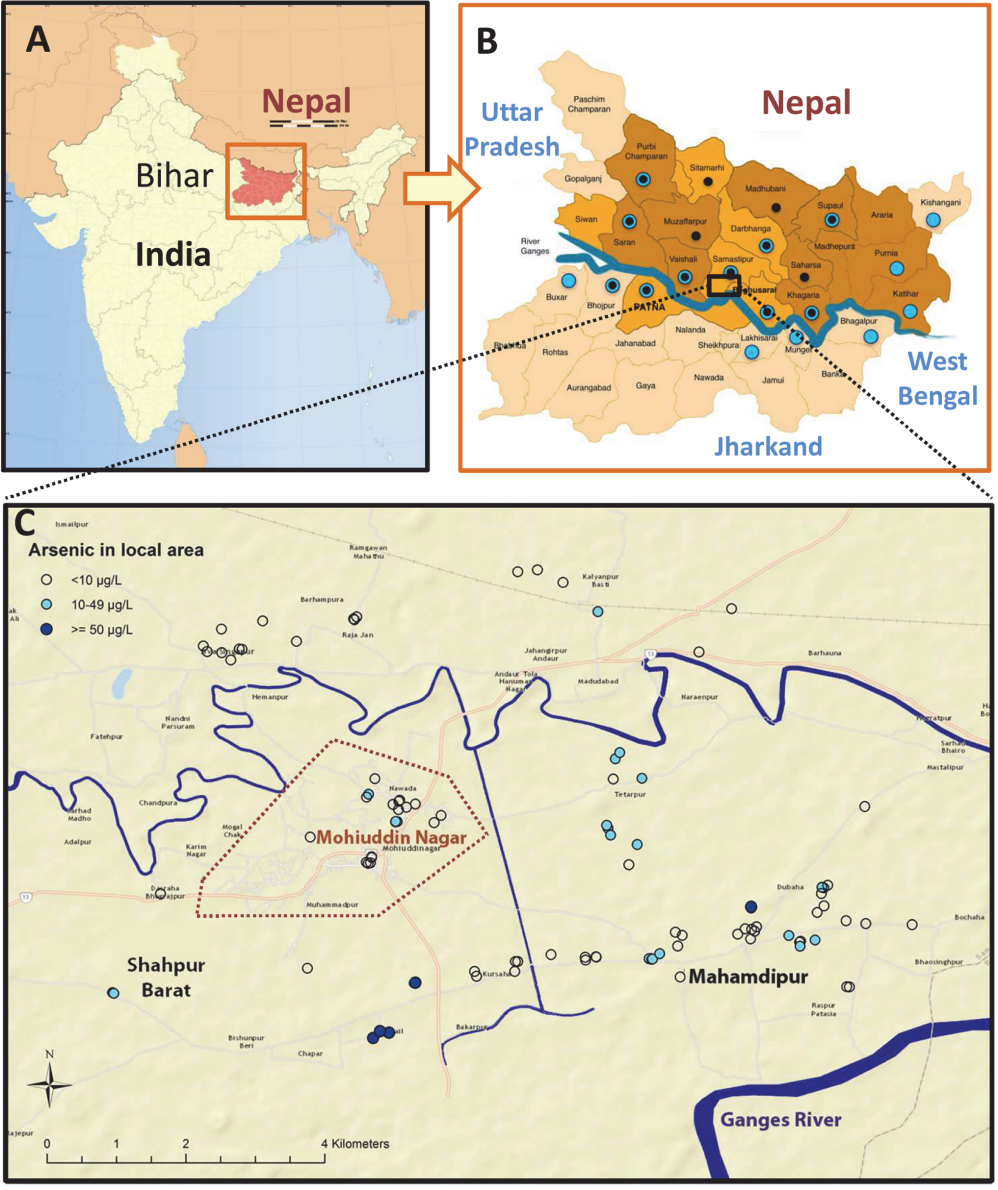
Map of study area. A. The State of Bihar, India. B. District level map of Bihar with study area boxed. Districts with blue dots have had arsenic contamination previously identified and those with black dots have reported antimonial resistance in clinical isolates [10]. C. Mohiuddin Nagar block: study area showing the town boundary (dotted line). Each dot represents the mean arsenic level determined from 5 wells in each study subject’s local area.

The 110 patients were aged between 3 and 60 years old, with a median age of 14 and a ratio of males to females of 3:2. Twenty percent of subjects lived within the area of Mohiuddin Nagar town (Fig. 2). Twenty-three (21%) of patients had experienced other illnesses prior to their VL episode including the infectious diseases of malaria (n = 2), tuberculosis (n = 2), hepatitis/jaundice (n = 4), cholera (n = 1) and polio (n = 1). Non-infectious illnesses included asthma (n = 7), diabetes (n = 1), arthritis (n = 1), eczema (n = 1) and neurological complaints (n = 3). All study subjects reported similar symptoms from their VL episode of fever, anorexia and weight loss (100%, 91%, 90% respectively) with malaise, abdominal distension and skin pigmentation being less common (72%, 44% and 7% respectively). Confirmation of the diagnosis of VL was available in the form of record of a positive rapid diagnostic test for VL (e.g. rK39 dipstick) in 26 subjects (24%). Parasitological confirmation was not available for any patient.

Seventy-one patients (65%) were only treated with SSG; 52 received one treatment, 18 received two and 1 patient received three SSG courses. Fourteen out of the 52 patients who received one treatment reported SSG treatment courses of 60 and 90 days duration which are 2 and 3 times the recommended duration. Thirty-nine patients (35%) were treated with miltefosine or amphotericin B before (n = 3) or after (n = 34) SSG treatment or both (n = 2). The mean number of treatments (using any drug) received per person was 1.6 (±0.6).

The number of clinical documents confirming the treatments administered was limited. For example, only 16 treatment records were available for review out of the 85 patients treated at the PHC. According to those records, the treatment of 7 out of 16 patients (44%) did not conform to WHO recommendations—either by dose (only 10mg/kg) or duration (20 vs 30d). However, the treatment duration reported by the patient in the questionnaire matched the PHC treatment card only in 10 out of these 16 patients (63%). Information on doses was not available from the patients’ interviews.

### Outcomes of antimonial treatment

Sixty two (56%) of the study subjects were classified as “treatment failure”: 10 (9%) died due to VL within 6 months, 40 (36%) patients experienced no clinical improvement, 8 (7%) patients relapsed and 4 (4%) terminated their treatment due to toxicity (Fig. 1). There was a high death rate in our cohort: 21 (19%) of 110 patients had died by the date of the interview. Sixteen (15%) of these patients died from VL, 6 of these deaths occurred after the 6 month follow up period for SSG treatment failure so they are not included in the primary outcome. One of these deaths was directly due to treatment toxicity. Five patients died from non-VL causes including HIV (n = 1), asthma (n = 1), liver failure (n = 1), paralysis (n = 1) and a road traffic accident (n = 1). Treatment outcomes were not recorded in the PHC register, PHC treatment cards or seldom in patient’s hand held documents.

### Arsenic exposure

The arsenic concentration in the wells tested ranged from < 3 μg/L up to 1050 μg/L arsenic. Fifty (44%) of the study subjects had at least one arsenic contaminated tube well in their home or surrounding area and 26 (24%) of them had a mean local arsenic level ≥ 10 μg/L (Table 1 and Fig. 2). Five (4.5%) patients had a mean local arsenic level of >50 μg/L, the previous Indian Government limit. Only 11 (10%) patients were aware of the issue of arsenic contamination and 8 of these lived in an area with local arsenic contamination. The remaining 18 out of the 26 patients living with local arsenic contamination had no knowledge of the issue.

For the cut off ≥10 μg/L arsenic the areas under the ROC curves were (1) 0.54 for treatment outcome, (2) 0.69 for all-cause mortality and (3) 0.65 for VL mortality. The sum of sensitivity and specificity was greater than 100% for all outcomes. The quality control assays showed a good agreement (Kappa = 90%, p<0.001) between the analysis performed at SOES and the University of Aberdeen laboratories.

### Treatment failure

**Fig 3:**
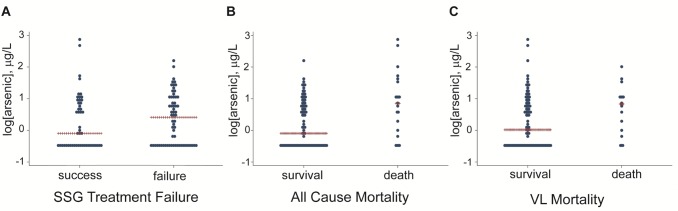
Dot plots of arsenic exposure and SSG treatment outcome and mortality in antimonial treated cohort (n = 110). Panels A, B and C show the log of the mean arsenic level plotted against outcome: SSG failure (MWU p = 0.42), all-cause mortality (MWU p = 0.005) and VL mortality (MWU p = 0.048). MWU = Mann Whitney U.

Although the median arsenic water exposure level was higher in patients with treatment failure (Fig. 3, panel A), this difference was not statistically significant (Mann Whitney U p = 0.42).

Only one covariate had a weak association with treatment failure (p<0.2) in the bivariate analysis: previous SSG treatment in the family (p = 0.11). This variable was not retained in the final multivariate model for lack of significance. Although the association between treatment duration and outcome was statistically significant (p = 0.01) this variable was not included in the final model as in the majority of cases the treatment duration is dependent on the outcome. The final logistic regression model only included the forced variables age, sex and location and shows a trend: patients with high mean local arsenic level have a higher risk of treatment failure (OR = 1.78) than patients using wells with arsenic concentration <10 μg/L, however this association is not statistically significant (95% CI: 0.7–4.6, p = 0.23).

### Mortality

As shown in the dot plots (Fig. 3, panels B and C) the median arsenic concentrations in water are significantly higher in the patients who died (group “all-cause mortality” (p = 0.005) and those who died due to VL (group “VL mortality” p = 0.048) compared to the patients that were still alive at the time of the field visits.

**Fig 4:**
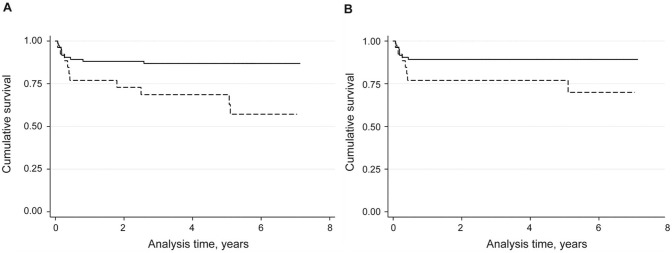
Kaplan Meier curves for all-cause and VL mortality. Panels A and B are survival curves comparing arsenic exposed (dotted line, = > 10 μg/L As) and non-arsenic exposed (solid line, <10 μg/L As) patients in the outcomes of all-cause mortality (log rank p = 0.004) and VL mortality (log rank p = 0.044).

The KM curves (Fig. 4, panels A and B) confirmed increased mortality in subjects exposed to elevated arsenic water levels (p = 0.004 and p = 0.044 for “all-cause” and “VL mortality,” respectively). Age was the only covariate to show a significant association with mortality and thus the final models only included the forced variables of age, sex and location. The multivariate Cox regression model showed that arsenic levels ≥ 10 μg/L increased significantly the risk of all-cause (HR 3.27; 95% CI: 1.4–8.1) and VL related (HR 2.65; 95% CI: 0.96–7.65) mortalities. However, the effect of arsenic exposure on all-cause mortality was found to vary with time. On the KM plots, the two survival lines representing arsenic exposed and non-exposed populations separate at 3 months where the effect of arsenic exposure becomes apparent. This was confirmed by a modified Cox regression model which allowed the effect of arsenic exposure to vary over time. No increased risk of mortality was detected with arsenic exposure during the first 3 months after start of VL symptoms. However, after 3 months, the effect of arsenic exposure significantly increased the risk of all-cause mortality (HR 8.56; 95% CI: 2.5–29.1) and of VL related mortality (HR 9.27; 95% CI: 1.8–49.0) (Table 2).

**Table 2:** Multivariate Cox regression analysis of effect elevated arsenic water levels (> = 10 μg/L) on risk of the outcomes of all-cause and VL related mortality in a cohort of SSG treated patients.

Variable	All-cause mortality		VL mortality	
	HR (95% CI)	p value	HR (95% CI)	p value
Effect of arsenic exposure*				
- during first 3 months from onset of VL symptoms	0.90 (0.19–4.37)	0.896	0.92 (0.19–4.53)	0.921
- after 3 months from onset of VL symptoms	8.56 (2.52–29.1)	0.001	9.27 (1.75–49.0)	0.009
**Possible Confounding Factors**				
**Age**				
0–5	Reference category		Reference category	
5–15	0.14 (0.04–0.47)	0.001	0.11 (0.03–0.45)	0.002
>15	0.19 (0.06–0.56)	0.003	0.17 (0.05–0.56)	0.003
**Sex**—female vs male	1.4 (0.53–3.67)	0.498	1.22 (0.41–3.61)	0.725
**Location**—outside of or within Mohiuddin Nagar town	1.04 (0.33–3.25)	0.951	1.30 (0.40–4.28)	0.662

HR = Hazard Ratio; CI = Confidence Interval

* Two time varying covariates were fitted in the model as the effect of arsenic exposure on mortality was found to vary with time

## Discussion

The results of this study found no significant relationship between exposure to arsenic and SSG treatment failure. However there was an unexpectedly high mortality rate in the cohort of SSG treated patients and a secondary analysis of mortality showed that arsenic exposure strongly increased the risk of both all-cause mortality and VL mortality.

The primary objective of this study was to evaluate if arsenic exposure is related to treatment failure in SSG treated patients. A positive correlation would have supported the laboratory finding that SSG resistant *Leishmania* parasites can be generated in a mouse model of oral arsenic exposure at environmentally relevant levels [14]. The results from this study, although they indicate a trend towards increased treatment failure in arsenic exposed patients, fail to confirm that this resistance generation mechanism is operating in the field. Analogous findings were obtained when the SSG treatment outcome was evaluated against the individual levels of arsenic in urine in the subset of patients where these were available (S1 Fig. and S1 Table in S1 Text). A number of reasons may explain why the field results do not support the laboratory results. Firstly, the numbers of patients available were limited as SSG treatment for VL is no longer recommended in Bihar and the study was underpowered to detect a low level of risk. Secondly, the in vivo experiments were performed in the upper range of arsenic levels found in Bihar. Only 2 patients in our study had arsenic levels in their local water supplies in this range and they both had a successful treatment with SSG. Thirdly, treatment failure from SSG is thought to be multifactorial and not only due to resistant parasites, particularly in that there is low correlation between in vitro resistance tests and clinical outcome [24]. Additionally, extensive work on SSG resistant parasites has shown that they have increased fitness and virulence when compared to SSG sensitive strains indicating that they would thus be preferentially transmitted and high levels of resistant parasites could be circulating [25]. These latter two reasons could mask any existing relationship between arsenic exposure and SSG treatment failure in VL.

Although multiple studies have been carried out on the mechanisms of antimonial resistance in the *Leishmania* parasite [9], only a few epidemiological studies have been performed looking at the clinical and demographic risk factors associated with SSG treatment failure in the leishmaniases [22,26]. The most relevant is a prospective study in Nepal on VL patients [22] that identified patients living on the border of Bihar as having a markedly increased chance of treatment failure. Additionally, fever over 12 weeks, interruption of treatment and ambulatory treatment were strong risk factors for treatment failure. All of the patients in our study received ambulatory treatment, treatment interruption was not specifically assessed and no association was found between prolonged fever and treatment failure. As arsenic contamination is an issue also in Nepal, it would be interesting to assess retrospectively the arsenic exposure in this cohort of patients. A study on rural VL care in Muzaffarpur district, Bihar [8] (northwest of the study area box in Fig. 2B) where no significant arsenic contamination has been detected [10], but SSG resistance is well established [1], had a SSG failure rate of 40% compared with 59% in our study. The difference in failure rates in these similar communities, with comparable district level literacy rates of 61.9% and 63.5% respectively [16] may be, among other factors, attributable to arsenic exposure and the increased mortality rate.

The main finding from our study, the strong relationship found between arsenic exposure and all-cause and VL mortality agrees with a population-based cohort study performed in Bangladesh [20] that demonstrated an increased risk for both all-cause mortality and infectious disease deaths with increasing arsenic exposure. The risk of death from VL in arsenic-exposed persons in our cohort is predominantly present greater than 3 months post the commencement of symptoms. This could be explained by 3 mechanisms: 1) the longer incubation time in arsenic exposed tissues would allow the parasites to generate arsenic tolerance and thus SSG resistance leading to ineffective treatment; 2) patients being infected with SSG-resistant parasites [17]; and 3) the risk of mortality increases with the progressive dampening effect of VL on the immune system [27] combined with the immunotoxicity of arsenic exposure [28]. The rural VL study in Muzaffarpur referred to above [8] only reported one death (0.7%) of undefined cause in a cohort of 138 (68 of whom were treated with SSG) compared with 21 deaths (19%) in our 110 patient cohort with 16 directly as a result of VL (14.5%). This could be explained by a number of factors as well as arsenic exposure such as: a different quality of primary health care between districts; a more virulent parasite population; or a general lower immune status, for example due to malnutrition, in our Samastipur study cohort. However, if the immunotoxicity of arsenic and VL combined is responsible for the increased death rate in our study then arsenic exposure may also increase mortality from VL with treatments other than SSG. This warrants further research.

Previous studies on mortality in VL have identified the extremes of age, (<5 and > 45 years old), long duration of symptoms, co-infections and laboratory abnormalities such as severe anaemia and jaundice as risk factors for death [29–32]. Due to the retrospective design of our study, and the rural management of the VL cases, information on the above clinical risk factors is mainly not available. Our data does agree with age associated risk at the extremes of age but no increased mortality risk was seen with delay to first SSG treatment.

It would have been ideal to perform a prospective study where the parasites’ sensitivity to pentavalent antimony in vitro in macrophages was correlated with arsenic exposure and the clinical outcome of SSG treatment. However, due to the high rate of treatment failure with antimony and the recommendation of discontinuation of use, this type of prospective study was not possible. The retrospective nature of the study meant that parasite isolates from antimony-treated cases were not available. The presence of SSG-resistant parasites was reported earlier in the Samastipur District [33]. Determining if antimonial resistant parasites are still circulating in the study area today may have helped to interpret the findings of our study, provided that stably resistant parasites had not extensively displaced SSG-sensitive isolates due to their increased fitness [34].

The retrospective design of this study also had some limitations in ascertaining VL cases and treatments used. No parasitological confirmation was available but most of the patients were diagnosed with VL by the rK39 rapid diagnostic test which shows a good specificity (90.6%) in the context of clinically suspected disease [35]. Relying on patient and relatives responses increased the risk of recall bias [36]. Therefore, the definition of treatment failure included the requirement for another treatment course which is more concrete that a subjective analysis of symptoms. As SSG treatment is prolonged and painful with a dramatic economic impact on the patient’s family, the recall bias for treatment may be limited. However, it is possible that administered doses were too low, or treatments were given with unrecalled interruptions that could have impacted on treatment outcome. Unfortunately, there was generally poor documentation of diagnostic methods, treatment duration and dosing. Finally, VL as a cause of death was confirmed by verbal autopsy only carried out by one physician. For this reason VL mortality is always presented with all-cause mortality.

A further substantial design difficulty in this study is the timing of the assessment of arsenic exposure. Forty percent of the patients had their tube well inserted in the years following their treatment episode and therefore a proxy of their arsenic exposure had to be created from the average of 5 wells surrounding their living area. Additionally, the level of arsenic contamination in a tube well water sample can vary according to prior usage that day, depth of well and age of well [37,38] adding further factors into the difficulty of building up an accurate picture of arsenic exposure at a historic time point. Although water arsenic levels were being collected on average 5 (± 1.3) years post treatment, it is generally assumed that arsenic in tube wells in the Bengal basin is either stable over time [20,39–41] or may rise gradually [37,38].

This cohort study was performed in an area where research on VL has not been undertaken previously and gives a view of practice in an area without any special intervention. It highlights the issue, previously identified in Muzaffarpur [8], that SSG was still being used in just under a quarter of patients treated between 2006 and 2010, with no record of its generally poor outcome, despite recommendations for its discontinuation since 2005 [42]. In the registers available at the PHC there was no indication of any treatment failure or relapse and, on interview, the staff were only aware of one death during the study period. The majority of patients (57%) felt the need to change health care provider to obtain an effective treatment. The doctors at the PHC knew of the issue of SSG resistance and have been aware of a drive to mainly use miltefosine or amphotericin but, worryingly, were unaware of the VL elimination programme. They were aware of the issue of arsenic contamination but unaware of its wide-ranging health effects. This work shows the urgent need for improved record keeping, education and intervention in both VL and arsenic contamination within these vulnerable communities [3,20].

A recent global review of the epidemiology of the leishmaniases gave an overall case fatality rate for VL of 10% [2] which is considerably lower than the VL mortality rate of 14.5% in our cohort. The study demonstrates that arsenic exposure is strongly associated with VL mortality in this antimony treated cohort and may be responsible for the elevated mortality rate. Unfortunately, the study was underpowered to confirm anything but a strong association between arsenic exposure and SSG treatment failure. This research into antimonial resistance and treatment failure has important implications for the leishmaniases worldwide and is a reminder to consider the environment in which an organism is propagating when assessing reasons for treatment failure and mortality. Further research into the relationship between arsenic exposure and VL mortality is required.

## Supporting Information

S1 Checklist STROBE Checklist. (DOCX)

S1 Text Arsenic levels in urine and antimonial treatment outcome in visceral leishmaniasis patients. (DOCX)

S1 Fig Dot plots of urinary arsenic exposure and SSG treatment outcome. Panels A and B show the log of the urinary arsenic level plotted against SSG treatment outcome: Urine biological samples (Panel A, MWU p = 0.81), urine with imputed values (Panel B, MWU p = 0.80). MWU = Mann Whitney U.
(TIF)
