# Supplementary material for: Arsenic Exposure and Outcomes of Antimonial Treatment in Visceral Leishmaniasis Patients in Bihar, India: A Retrospective Cohort Study
Source: PLoS Negl Trop Dis. 2015 Mar 2;9(3):e0003518. doi: 10.1371/journal.pntd.0003518 (PMC4346263; doi:10.1371/journal.pntd.0003518)
Supplement: S1 Checklist — (DOCX) [file pntd.0003518.s001.docx]

STROBE Statement—checklist of items that should be included in reports of observational studies

|  | Item No. | Recommendation | | Page  No. | Relevant text from manuscript |
| --- | --- | --- | --- | --- | --- |
| **Title and abstract** | 1 | (*a*) Indicate the study’s design with a commonly used term in the title or the abstract | | 1 | “A retrospective cohort study” |
|  |  | (*b*) Provide in the abstract an informative and balanced summary of what was done and what was found | | 2 | Abstract |
| Introduction | | | | |  |
| Background/rationale | 2 | Explain the scientific background and rationale for the investigation being reported | | 4,5 | “It is thus possible that parasites were exposed to accumulated arsenic in the liver and spleen of populations drinking arsenic-contaminated water, and became cross-resistant to antimony through mechanisms developed to deal with arsenic. Proof of concept of this hypothesis has been established in our laboratory by exposing mice to environmentally relevant levels of trivalent arsenic in their drinking water and demonstrating diminished efficacy of the pentavalent antimonial compound SSG *in vivo* and, in an *in vitro* macrophage assay, that *ex vivo* amastigotes from these arsenic exposed mice were resistant to 500 μg/ml SSG [14]*.*” |
| Objectives | 3 | State specific objectives, including any prespecified hypotheses | | 5 | “we sought to test the hypothesis that arsenic exposure increases the risk of VL treatment failure with antimonial compounds in the field setting” |
| Methods | | | | |  |
| Study design | 4 | Present key elements of study design early in the paper | 6,7 | | “Retrospective cohort study”, “treatment failure as a proxy for parasite resistance” “questionnaire” |
| Setting | 5 | Describe the setting, locations, and relevant dates, including periods of recruitment, exposure, follow-up, and data collection | 6,7 | | “Mohiuddin Nagar”, “2006-2010”, “questionnaire” “Clinical records, either the patient’s own documents or those held at the PHC” |
| Participants | 6 | (*a*) *Cohort study*—Give the eligibility criteria, and the sources and methods of selection of participants. Describe methods of follow-up  *Case-control study*—Give the eligibility criteria, and the sources and methods of case ascertainment and control selection. Give the rationale for the choice of cases and controls  *Cross-sectional study*—Give the eligibility criteria, and the sources and methods of selection of participants | 6,7 | | “PHC register” “identified in the visited villages” “included in the study if they gave a clear history of having been treated with SSG (see below) and at least one of the SSG treatments fell inside the study recruitment period (e.g. 2006 to 2010). Patients were excluded if they did not receive SSG treatment or if their SSG treatment was terminated due to unavailability of SSG.” |
|  |  | (*b*) *Cohort study*—For matched studies, give matching criteria and number of exposed and unexposed  *Case-control study*—For matched studies, give matching criteria and the number of controls per case |  | |  |
| Variables | 7 | Clearly define all outcomes, exposures, predictors, potential confounders, and effect modifiers. Give diagnostic criteria, if applicable | 8 | | “Patients were classified into the following treatment outcome categories: success, no clinical improvement, relapse, death and toxicity taking 6-month cut off time from end of treatment as reference, in accordance with WHO guidance and VL clinical trial protocols [1]. ‘Treatment success’ was defined as patients who received SSG treatment and had not required another VL treatment within 6 months. ‘No clinical improvement’ were patients who experienced no change or a worsening of their original symptoms during or by the end of a full course of treatment who required a further VL treatment. This subgroup included patients who reported a treatment course of 60 days or more which is 2 times the recommended SSG treatment duration. ‘Relapse’ were patients who experienced a return of signs or symptoms of VL, for which they required further treatment, after initially having experienced a return to health following a course of treatment. If the outcome was ‘Death’, a verbal autopsy was carried out by the physician on the field team to ascertain if the subject died directly as a result of VL. ‘Toxicity’ were patients whose treatment was terminated due to intolerable side effects. The term ‘treatment failure’ in this study covers all adverse outcomes that occurred within 6 months of VL treatment: no clinical improvement, relapse, death due to VL and toxicity.” |
| Data sources/ measurement | 8* | For each variable of interest, give sources of data and details of methods of assessment (measurement). Describe comparability of assessment methods if there is more than one group | 8,9 | | “The average arsenic concentration of the patient’s main water source and 4 tube wells surrounding the patient’s home was taken as an indication of the level of arsenic exposure for the patient”. “atomic absorption spectrometry (FI-HG-AAS) was used to quantify the total arsenic content.” |
| Bias | 9 | Describe any efforts to address potential sources of bias | 18 | | “Relying on patient and relatives responses increased the risk of recall bias [36]. Therefore, the definition of treatment failure included the requirement for another treatment course which is more concrete that a subjective analysis of symptoms.” |
| Study size | 10 | Explain how the study size was arrived at | 6,7 | | “potential SSG treated subjects were identified from the VL patient register at the Primary Health Centre (PHC) in Mohiuddin Nagar and were searched for in their listed villages, at least twice, for inclusion in the study. Second, additional VL patients were identified in the visited villages.” |

Continued on next page

| Quantitative variables | 11 | Explain how quantitative variables were handled in the analyses. If applicable, describe which groupings were chosen and why | 9 | “The average arsenic concentration in the 5 tube wells was dichotomized using the WHO threshold for arsenic (As) in water: >= 10 μg/L.” “Briefly, age was split into 3 categories (e.g. < 5, 6-15 and > 16 years old) and SSG treated patients were dichotomized based on their “treatment course” using the duration of 30 days recommended by WHO [5].” “the “time to treatment” (e.g. < 12 weeks and ≥ 12 weeks in accordance with previous literature [22])” |
| --- | --- | --- | --- | --- |
| Statistical methods | 12 | (*a*) Describe all statistical methods, including those used to control for confounding | 9-11 | Described in paragraphs under heading “Statistical analysis” |
|  |  | (*b*) Describe any methods used to examine subgroups and interactions | n/a |  |
|  |  | (*c*) Explain how missing data were addressed | SI | How the missing data on biological samples were dealt with is described in “Methods” section of SI |
|  |  | (*d*) *Cohort study*—If applicable, explain how loss to follow-up was addressed  *Case-control study*—If applicable, explain how matching of cases and controls was addressed  *Cross-sectional study*—If applicable, describe analytical methods taking account of sampling strategy | n/a |  |
|  |  | (*e*) Describe any sensitivity analyses |  |  |
| Results | | | | |
| Participants | 13* | (a) Report numbers of individuals at each stage of study—eg numbers potentially eligible, examined for eligibility, confirmed eligible, included in the study, completing follow-up, and analysed | 12 | “We identified 606 patients treated for VL from January 1st 2006 to December 31st 2010 at the PHC of Mohiuddin Nagar. One hundred and thirty four (22%) of them were treated with SSG. The rest (n=472) were excluded from the study as they received miltefosine or amphotericin B (n=470) or were duplicate entries (n=2). Twenty-five additional VL patients treated with SSG from 2006 to 2010 were identified at the time of the fieldwork visits. Out of the 159 SSG treated patients identified, the house of 113 (71%) of them was located. The reasons for being unable to locate patients included incomplete/inaccurate information (n=43) and distance from Mohiuddin Nagar block (n=3). From the 113 patients, 69 (61%) of them were present for interview. Twenty one (19%) of the subjects were dead and 23 (20%) were living outside the study area at the time of the visit. Relatives from the 44 missing subjects were interviewed. Based on the information gathered, 3 patients were excluded as they had their SSG treatment terminated due to unavailability of the drug. A cohort of 110 VL patients treated with SSG was finally included in the analyses (Figure 1).” |
|  |  | (b) Give reasons for non-participation at each stage | n/a |  |
|  |  | (c) Consider use of a flow diagram | Figure 1 |  |
| Descriptive data | 14* | (a) Give characteristics of study participants (eg demographic, clinical, social) and information on exposures and potential confounders | 12, 13, 14 | Under headings “VL cases and treatment” and “Arsenic exposure” |
|  |  | (b) Indicate number of participants with missing data for each variable of interest | SI | “Of the 69 patients where biological samples were available, only 66 were in the final cohort of 110 and one sample was unable to be analyzed, therefore arsenic exposure in urine was only able to be assessed on an individual basis in 65 (59%) study subjects.” |
|  |  | (c) *Cohort study*—Summarise follow-up time (eg, average and total amount) | Not stated | Retrospective, different endpoints |
| Outcome data | 15* | *Cohort study*—Report numbers of outcome events or summary measures over time | 13 | “Sixty two (56%) of the study subjects were classified as “treatment failure”: 10 (9%) died due to VL within 6 months, 40 (36%) patients experienced no clinical improvement, 8 (7%) patients relapsed and 4 (4%) terminated their treatment due to toxicity (Figure 1). There was a high death rate in our cohort: 21 (19 %) of 110 patients had died by the date of the interview. Sixteen (15%) of these patients died from VL, 6 of these deaths occurred after the 6 month follow up period for SSG treatment failure so they are not included in the primary outcome. One of these deaths was directly due to treatment toxicity. Five patients died from non-VL causes including HIV (n=1), asthma (n=1), liver failure (n=1), paralysis (n=1) and a road traffic accident (n=1). Treatment outcomes were not recorded in the PHC register, PHC treatment cards or seldom in patient’s hand held documents. “ |
|  |  | *Case-control study—*Report numbers in each exposure category, or summary measures of exposure | n/a |  |
|  |  | *Cross-sectional study—*Report numbers of outcome events or summary measures | n/a |  |
| Main results | 16 | (*a*) Give unadjusted estimates and, if applicable, confounder-adjusted estimates and their precision (eg, 95% confidence interval). Make clear which confounders were adjusted for and why they were included | 14, 15 | Under headings “Treatment failure” and “Mortality” |
|  |  | (*b*) Report category boundaries when continuous variables were categorized | Table 1 |  |
|  |  | (*c*) If relevant, consider translating estimates of relative risk into absolute risk for a meaningful time period | n/a |  |

Continued on next page

| Other analyses | 17 | Report other analyses done—eg analyses of subgroups and interactions, and sensitivity analyses | n/a |  |
| --- | --- | --- | --- | --- |
| Discussion | | | | |
| Key results | 18 | Summarise key results with reference to study objectives | 16 | “The results of this study found no significant relationship between exposure to arsenic and SSG treatment failure. However there was an unexpectedly high mortality rate in the cohort of SSG treated patients and a secondary analysis of mortality showed that arsenic exposure strongly increased the risk of both all-cause mortality and VL mortality.” |
| Limitations | 19 | Discuss limitations of the study, taking into account sources of potential bias or imprecision. Discuss both direction and magnitude of any potential bias | 18,19, | “It would have been ideal to perform a prospective study where the parasites’ sensitivity to pentavalent antimony *in vitro* in macrophages was correlated with arsenic exposure and the clinical outcome of SSG treatment. However, due to the high rate of treatment failure with antimony and the recommendation of discontinuation of use, this type of prospective study was not possible. The retrospective nature of the study meant that parasite isolates from antimony-treated cases were not available. The presence of SSG-resistant parasites was reported earlier in the Samastipur District [33]. Determining if antimonial resistant parasites are still circulating in the study area today may have helped to interpret the findings of our study, provided that stably resistant parasites had not extensively displaced SSG-sensitive isolates due to their increased fitness [34].  The retrospective design of this study also had some limitations in ascertaining VL cases and treatments used. No parasitological confirmation was available but most of the patients were diagnosed with VL by the rK39 rapid diagnostic test which shows a good specificity (90.6%) in the context of clinically suspected disease [35]. Relying on patient and relatives responses increased the risk of recall bias [36]. Therefore, the definition of treatment failure included the requirement for another treatment course which is more concrete that a subjective analysis of symptoms. As SSG treatment is prolonged and painful with a dramatic economic impact on the patient’s family, the recall bias for treatment may be limited. However, it is possible that administered doses were too low, or treatments were given with unrecalled interruptions that could have impacted on treatment outcome. Unfortunately, there was generally poor documentation of diagnostic methods, treatment duration and dosing. Finally, VL as a cause of death was confirmed by verbal autopsy only carried out by one physician. For this reason VL mortality is always presented with all-cause mortality.  A further substantial design difficulty in this study is the timing of the assessment of arsenic exposure. Forty percent of the patients had their tube well inserted in the years following their treatment episode and therefore a proxy of their arsenic exposure had to be created from the average of 5 wells surrounding their living area. Additionally, the level of arsenic contamination in a tube well water sample can vary according to prior usage that day, depth of well and age of well [37,38] adding further factors into the difficulty of building up an accurate picture of arsenic exposure at a historic time point. Although water arsenic levels were being collected on average 5 (± 1.3) years post treatment, it is generally assumed that arsenic in tube wells in the Bengal basin is either stable over time [20,39–41] or may rise gradually [37,38].” |
| Interpretation | 20 | Give a cautious overall interpretation of results considering objectives, limitations, multiplicity of analyses, results from similar studies, and other relevant evidence | 16,17 | “The primary objective of this study was to evaluate if arsenic exposure is related to treatment failure in SSG treated patients. A positive correlation would have supported the laboratory finding that SSG resistant *Leishmania* parasites can be generated in a mouse model of oral arsenic exposure at environmentally relevant levels [14]. The results from this study, although they indicate a trend towards increased treatment failure in arsenic exposed patients, fail to confirm that this resistance generation mechanism is operating in the field. Analogous findings were obtained when the SSG treatment outcome was evaluated against the individual levels of arsenic in urine in the subset of patients where these were available (Supplementary material). A number of reasons may explain why the field results do not support the laboratory results. Firstly, the numbers of patients available were limited as SSG treatment for VL is no longer recommended in Bihar and the study was underpowered to detect a low level of risk. Secondly, the *in vivo* experiments were performed in the upper range of arsenic levels found in Bihar. Only 2 patients in our study had arsenic levels in their local water supplies in this range and they both had a successful treatment with SSG. Thirdly, treatment failure from SSG is thought to be multifactorial and not only due to resistant parasites, particularly in that there is low correlation between *in vitro* resistance tests and clinical outcome [24]. Additionally, extensive work on SSG resistant parasites has shown that they have increased fitness and virulence when compared to SSG sensitive strains indicating that they would thus be preferentially transmitted and high levels of resistant parasites could be circulating [25]. These latter two reasons could mask any existing relationship between arsenic exposure and SSG treatment failure in VL.”  “The main finding from our study, the strong relationship found between arsenic exposure and all-cause and VL mortality agrees with a population-based cohort study performed in Bangladesh [20] that demonstrated an increased risk for both all-cause mortality and infectious disease deaths with increasing arsenic exposure. The risk of death from VL in arsenic-exposed persons in our cohort is predominantly present greater than 3 months post the commencement of symptoms. This could be explained by 3 mechanisms: 1) the longer incubation time in arsenic exposed tissues would allow the parasites to generate arsenic tolerance and thus SSG resistance leading to ineffective treatment; 2) patients being infected with SSG-resistant parasites [17]; and 3) the risk of mortality increases with the progressive dampening effect of VL on the immune system [27] combined with the immunotoxicity of arsenic exposure [28].” |
| Generalisability | 21 | Discuss the generalisability (external validity) of the study results | 17, 18 | “Although multiple studies have been carried out on the mechanisms of antimonial resistance in the *Leishmania* parasite [9], only a few epidemiological studies have been performed looking at the clinical and demographic risk factors associated with SSG treatment failure in the leishmaniases [22,26]. The most relevant is a prospective study in Nepal on VL patients [22] that identified patients living on the border of Bihar as having a markedly increased chance of treatment failure. Additionally, fever over 12 weeks, interruption of treatment and ambulatory treatment were strong risk factors for treatment failure. All of the patients in our study received ambulatory treatment, treatment interruption was not specifically assessed and no association was found between prolonged fever and treatment failure. As arsenic contamination is an issue also in Nepal, it would be interesting to assess retrospectively the arsenic exposure in this cohort of patients. A study on rural VL care in Muzaffarpur district, Bihar [8] (northwest of the study area box in Figure 2B) where no significant arsenic contamination has been detected [10], but SSG resistance is well established [1], had a SSG failure rate of 40% compared with 59% in our study. The difference in failure rates in these similar communities, with comparable district level literacy rates of 61.9% and 63.5% respectively [16] may be, among other factors, attributable to arsenic exposure and the increased mortality rate.”  “The rural VL study in Muzaffarpur referred to above [8] only reported one death (0.7%) of undefined cause in a cohort of 138 (68 of whom were treated with SSG) compared with 21 deaths (19%) in our 110 patient cohort with 16 directly as a result of VL (14.5%). This could be explained by a number of factors as well as arsenic exposure such as: a different quality of primary health care between districts; a more virulent parasite population; or a general lower immune status, for example due to malnutrition, in our Samastipur study cohort. However, if the immunotoxicity of arsenic and VL combined is responsible for the increased death rate in our study then arsenic exposure may also increase mortality from VL with treatments other than SSG. This warrants further research.  Previous studies on mortality in VL have identified the extremes of age, (<5 and > 45 years old), long duration of symptoms, co-infections and laboratory abnormalities such as severe anaemia and jaundice as risk factors for death [29–32]. Due to the retrospective design of our study, and the rural management of the VL cases, information on the above clinical risk factors is mainly not available. Our data does agree with age associated risk at the extremes of age but no increased mortality risk was seen with delay to first SSG treatment.” |
| Other information | |  | | |
| Funding | 22 | Give the source of funding and the role of the funders for the present study and, if applicable, for the original study on which the present article is based |  | “This work was supported by a Clinical PhD Fellowship to MRP (090665) and a Principal Research Fellowship to AHF (079838) from the Wellcome Trust. “ |

*Give information separately for cases and controls in case-control studies and, if applicable, for exposed and unexposed groups in cohort and cross-sectional studies.

**Note:** An Explanation and Elaboration article discusses each checklist item and gives methodological background and published examples of transparent reporting. The STROBE checklist is best used in conjunction with this article (freely available on the Web sites of PLoS Medicine at http://www.plosmedicine.org/, Annals of Internal Medicine at http://www.annals.org/, and Epidemiology at http://www.epidem.com/). Information on the STROBE Initiative is available at www.strobe-statement.org.
