# Supplementary material for: Arsenic Exposure and Outcomes of Antimonial Treatment in Visceral Leishmaniasis Patients in Bihar, India: A Retrospective Cohort Study
Source: PLoS Negl Trop Dis. 2015 Mar 2;9(3):e0003518. doi: 10.1371/journal.pntd.0003518 (PMC4346263; doi:10.1371/journal.pntd.0003518)
Supplement: S1 Text — (DOCX) [file pntd.0003518.s002.docx]

**Supporting Information**

**Arsenic levels in urine and antimonial treatment outcome in visceral leishmaniasis patients**

**Methods**

Sample collection and analysis

At the time of field questionnaire patients were requested to provide a sample of urine of at least 5 ml. Urine samples were preserved with a drop of hydrochloric acid and kept in cold conditions.

Prior to analysis, the urine samples were centrifuged at 3000 rev min^-1^ for 10 min and filtered through a 0.45-μm membrane filter (Chromatodisc 25A) to remove the suspended particulate. The urine was then digested for total arsenic analysis: 1 ml of the urine sample was placed in a small Kjehldahl flask and to this was added Suprapur^®^ acid (0.5 ml HNO_3_, 0.2 ml H_2_SO_4_ and 0.2 ml HC1O_4_). The sample was heated using a small funnel at the top of a sand-bath until it became clear. If necessary, a 0.2 ml aliquot of HNO_3_ was subsequently added. Again the sample was heated until fumes of SO_3_ evolved. The solution was cooled and made up to a volume of 5 ml. Total arsenic in the urine samples, as stable urinary metabolites of arsenic, was measured using FI-HG-AAS against a standard reference material (SRM 2670) obtained from the National Institute of Standards and Technology (NIST, Gaithersburg, MD, USA).

For quality control, 18 (26%) of the available urine samples were selected to represent normal (n=9; 5-40 μg/L) and elevated (n=9; >40 μg/L) urinary arsenic levels detected. These samples were re-analyzed by Inductively Coupled Plasma Mass Spectrometry (ICP-MS) at Aberdeen University using a method described previously [1].

Data management

The urine arsenic exposure variable was created with a binary cut off of arsenic contamination for a spot urine sample above the normal range of 5-40 μg/L [2,3]. In patients for whom it was not been possible to obtain a biological sample, an imputed value for their urinary arsenic level was calculated, using the arsenic water level variable described in the main text, in a regression prediction model controlling for age, sex and geographical location.

Statistical Analysis

Dot plots of the log of urinary arsenic level and Mann Whitney U tests were used to describe the relationship between urinary arsenic level, as a continuous variable, and SSG treatment outcome (Figure S1). Receiver Operating Characteristic (ROC) curves were used to evaluate the cut off for arsenic in urine (40 μg/L) against the outcome of SSG treatment. The Kappa index was used to evaluate the agreement between the arsenic levels reported by the two laboratories (SOES and Aberdeen University) that analyzed the quality control samples.

A logistic regression model was employed to assess if elevated urinary arsenic increased the risk of treatment failure in SSG treated patients. The analysis of covariate against outcome using logistic regression is presented in Table 1 in the main text. The final regression model included arsenic exposure, three a priori selected variables (“forced” variables”: age, sex and location) and any variables that were associated with mortality as assessed by the log rank test (p <0.2) and remained significant (p <0.05) in the final model. The results were presented as odds ratios (OR) and their 95% Confidence Intervals (CI).

Mortality analysis was not performed using urinary arsenic values as there were no direct measurements of urinary arsenic in deceased patients.

**Results**

Of the 69 patients where biological samples were available, only 66 were in the final cohort of 110 and one sample was unable to be analyzed, therefore arsenic exposure in urine was only able to be assessed on an individual basis in 65 (59%) study subjects.

The correlation between arsenic in urine and in the water in the study subject’s local environment gives an R-squared value of 0.60 (P < 0.0001). From this relationship 45 urinary values were imputed to allow analysis of the full cohort. All the results for urinary arsenic analysis will be presented for 2 datasets: biological samples only (n=65) and with the imputed values (n=110).

For the cut off >40 μg/L urinary arsenic the areas under the ROC curves were 0.53 and 0.49 for the two datasets respectively. The sum of sensitivity and specificity was ≥100 % for both datasets. The quality control assays showed a moderate agreement (Kappa=72%, p=0.012) between the analysis performed at SOES and the University of Aberdeen laboratories.

The median arsenic exposure values were similar in patients who failed and had a successful treatment with SSG (Figure S1, A and B). The covariate of previous SSG treatment in the family with a weak association with treatment failure (p=0.103) was initially included in a multivariate model. The final logistic regression model only included the forced variables age, sex and location. When analyzing the biological samples only a trend was observed: patients with elevated urinary arsenic have a higher risk of treatment failure (OR 2.06) than those with an arsenic level within the normal range, however this association is not statistically significant (95% CI: 0.57-7.47, p=0.269). When using the data set that includes imputed values, the risk of treatment failure with elevated urinary arsenic was markedly attenuated (OR 1.29) and the association became less significant (95% CI: 0.53-3.19, p=0.575) (Table S1).

In the multivariate analysis using biological samples only, being of an older age carried a significant risk when adjusted for urine arsenic exposure level, location and sex. This risk disappears when the imputed data is also used in the analysis: all the patients who were not available for biological sample collection due to relocation were older than 5 years old at their time of treatment and 66.7% of this group had a successful 1^st^ treatment with SSG (compared with a 43.6% success rate in the full cohort).

**Discussion**

Multivariate analysis shows a non-significant trend of increased risk of treatment failure with elevated arsenic levels. Measurement of arsenic levels in urine is an excellent biomarker of arsenic exposure [4] and allows for a direct individual assessment which includes any exposure from foods and other sources. However in the context of this study it has many disadvantages. Urine represents arsenic exposure in the preceding 24 h [5] where we are interested in representing arsenic exposure at the time of the patient’s VL episode up to 6 years prior to the study. This, coupled with the need to impute 41% (n=45) values to be able to analyze the full the cohort, due to the unavailability of these VL patients, decreases the value of urine as an exposure variable.

Additionally there is controversy on how to present urine measurements. The ideal way to measure arsenic in urine is through a 24 h collection but this is often impractical in field study settings. Some studies use μg/g of creatinine to address this but arsenic exposure has been shown to increase creatinine clearance which would distort results [6].

There is no definitive cut off for elevated arsenic in urine. In 2001, D.N.G. Mazumder recommended a level of > 50 μg/L urinary arsenic [7], but this was to diagnose ‘arsenicosis’ and is coupled with drinking water at >50 μg/L, levels under which are relevant in this study (see main text). In view of this, a cut off of urinary arsenic levels above the normal range of 5- 40 μg/L [2,3] was utilized in this study.

**Table S1**: **Multivariate logistic regression analysis using urinary arsenic data**

|  | **Urine biological samples**  **n=65** | | **Urine with imputed values n=110** | |
| --- | --- | --- | --- | --- |
| **Variable** | OR (95% C.I.) | p value | OR (95% C.I.) | p value |
| **Urine arsenic level**  > 40 μg/L | 2.06 (0.57-7.47) | 0.269 | 1.29 (0.53-3.19) | 0.575 |
| **Possible confounding factors** |  |  |  |  |
| **Age (years)** |  |  |  |  |
| 0-5 | Ref. cat |  | Ref. cat |  |
| 6-15 | 9.57 (1.36-67.2) | 0.023 | 1.17 (0.34-4.10) | 0.798 |
| > 15 | 6.95 (1.03-46.9) | 0.047 | 0.86 (0.25-2.94) | 0.808 |
| **Sex**  Female vs male | 0.62 (0.20-1.96) | 0.419 | 0.8 (0.36-1.81) | 0.595 |
| **Location**  Outside of or within  Mohiuddin Nagar town | 0.97 (0.23-4.14) | 0.964 | 1.32 (0.49-3.57) | 0.582 |

OR = Odds Ratio, CI = Confidence Interval. Ref. cat = Reference category

**References S1**

1. Williams PN, Villada A, Deacon C, Raab A, Figuerola J, Green AJ, Feldmann J, Meharg AA (2007) Greatly enhanced arsenic shoot assimilation in rice leads to elevated grain levels compared to wheat and barley. Environ Sci Technol 41: 6854-6859.

2. Ravenscroft, P., Brammer, H., and Richards, K. (2009) Arsenic Pollution: A Global Synthesis. Chichester: Wiley-Blackwell. 588 p.

3. Mandal BK, Suzuki KT (2002) Arsenic round the world: a review. Talanta 58: 201-235.

4. Concha G, Nermell B, Vahter M (2006) Spatial and temporal variations in arsenic exposure via drinking-water in Northern Argentina. Journal of Health Population and Nutrition 24: 317-326.

5. Vahter M (1994) What are the chemical forms of arsenic in urine, and what can they tell us about exposure? Clin Chem 40: 679-680.

6. Gamble MV, Liu X (2005) Urinary creatinine and arsenic metabolism. Environ Health Perspect 113: A442-A443.

7. Mazumder DN (2001) Clinical aspects of chronic arsenic toxicity. J Assoc Physicians India 49: 650-655.
