# Supplementary figures and images for: Arsenic Exposure and Outcomes of Antimonial Treatment in Visceral Leishmaniasis Patients in Bihar, India: A Retrospective Cohort Study
Source: PLoS Negl Trop Dis. 2015 Mar 2;9(3):e0003518. doi: 10.1371/journal.pntd.0003518 (PMC4346263; doi:10.1371/journal.pntd.0003518)

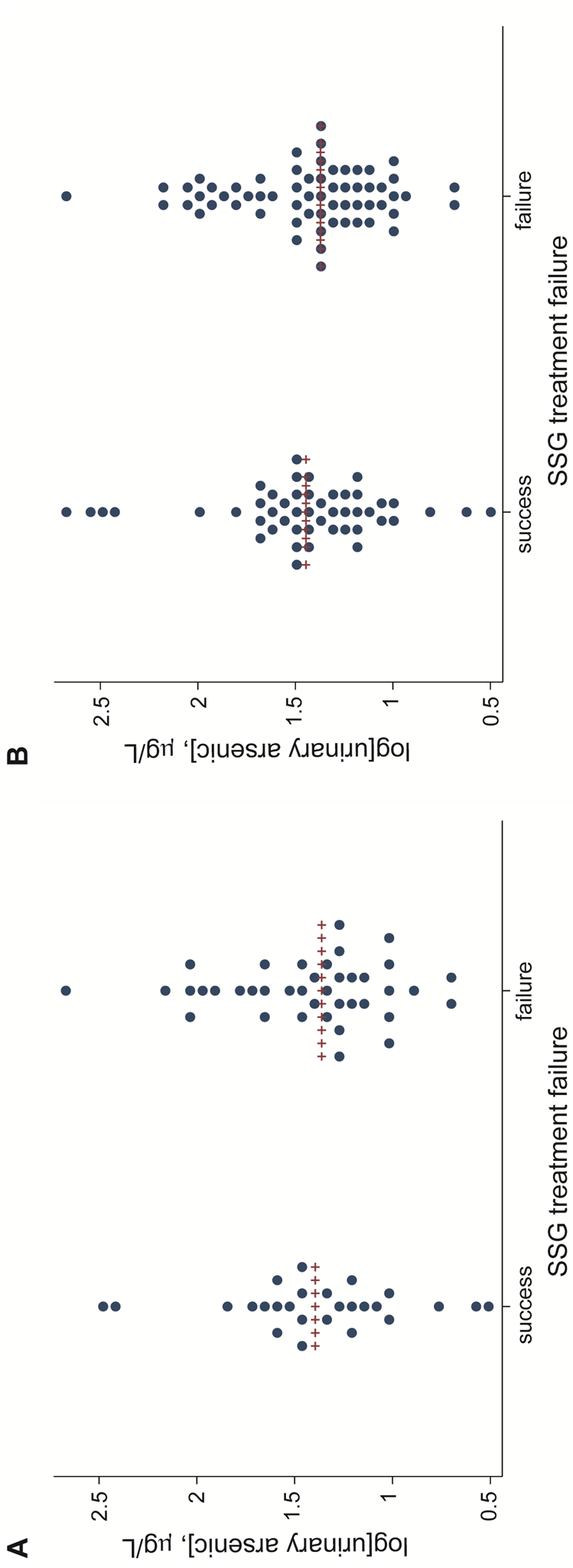

Supplement: S1 Fig — Panels A and B show the log of the urinary arsenic level plotted against SSG treatment outcome: Urine biological samples (Panel A, MWU p = 0.81), urine with imputed values (Panel B, MWU p = 0.80). MWU = Mann Whitney U. (TIF) [file pntd.0003518.s003.tif]
